# The Potential Role of Hypochlorhydria in the Development of Duodenal Dysbiosis: A Preliminary Report

**DOI:** 10.3389/fcimb.2022.854904

**Published:** 2022-04-19

**Authors:** Simone Filardo, Giulia Scalese, Camilla Virili, Stefano Pontone, Marisa Di Pietro, Antonio Covelli, Giorgio Bedetti, Paride Marinelli, Giovanni Bruno, Ilaria Stramazzo, Marco Centanni, Rosa Sessa, Carola Severi

**Affiliations:** ^1^ Department of Public Health and Infectious Diseases, University of Rome “Sapienza”, Rome, Italy; ^2^ Department of Translational and Precision Medicine, University of Rome “Sapienza”, Rome, Italy; ^3^ Department of Medico-surgical Sciences and Biotechnologies, University of Rome “Sapienza”, Latina, Italy; ^4^ Department of Surgical Sciences, University of Rome “Sapienza”, Rome, Italy; ^5^ Endocrine Unit, Azienda Unità Sanitaria Locale (AUSL) Latina, Latina, Italy

**Keywords:** microbiota, duodenal dysbiosis, intragastric pH, chronic atrophic gastritis, nonatrophic pangastritis, metagenomic analysis

## Abstract

In recent years, the role of gastric and duodenal microbiota has acquired increasing importance in the homeostasis of the host, although, to date, most evidence concern the faecal microbiota. Indeed, the gastric, and duodenal microbiota are challenging to study, due to gastric acid, bile, digestive enzymes, and rapid transit time. Specifically, the gastric acid environment may influence their bacterial composition since the acid barrier protects against orally ingested microorganisms and leads to their inactivation before reaching the intestine. The aim of this study was to assess a correlation between intragastric pH and gastric as well as intestinal microbiota of patients with histologic gastric alterations. pH was measured in the gastric juice and the bacterial composition in gastric and duodenal biopsies and faecal samples, was investigated *via* 16s rRNA gene sequencing. The main result is the direct correlation of duodenal microbiota biodiversity, *via* alpha diversity measures, with intragastric pH values. In particular, patients with hypochlorhydria showed increased duodenal microbiota biodiversity, higher intragastric pH values being prevalent in patients with chronic atrophic gastritis. Lastly, the latter was also strongly associated to the presence of oral bacteria, like *Rothia mucilaginosa, Streptococcus salivarius* and *Granulicatella adiacens*, in the duodenal microbiota. In conclusions, our results suggest a low-acid gastric environment as a contributive factor for duodenal dysbiosis, potentially leading to the development of pathological conditions of the gastrointestinal tract.

## 1. Introduction

Human gut microbiota (GM) is made of a vast number of microorganisms which colonize the digestive tract, including bacteria, archaea, fungi, and viruses (Tziatzios et al., 2020). In the last decades several efforts were made to study and understand GM functions. Nowadays it is well-known that GM plays a central role in maintaining the homeostasis of the host, acting as an effective and highly specialized barrier against pathogens, interacting with the immune system and contributing to the fermentative process of dietary and endogenous substrates (Tziatzios et al., 2020). Currently, the existence of a resident gastric microbiota, whose functions are still to be understood, has been widely demonstrated. Indeed, gastric microbiota evaluation results so challenging to study for the low pH, the difficulty of obtaining representative samples of the entire gastric mucosa, and the potential contamination by oral microorganisms, which can lead to the distortion of the original gastric microbial composition (Conti et al., 2020).

Beyond the gastric microbiota, also the duodenal one is challenging to study due to the combination of gastric acid, bile, digestive enzymes, and rapid transit time. All these conditions make the duodenum another hostile environment for bacterial growth, and this results in a lower bacterial abundance compared with the others gut sites, like jejunum, ileum, and colon. Nevertheless, since commensal duodenal microbiota plays an essential role in nutrient acquisition, resistance to pathogen colonization, immune development, and epithelial barrier function (Wauters et al., 2020), it is nowadays fundamental to study and evaluate its composition as well. Although it is not frequently addressed in the literature, it appears that the gastric and duodenal microbiota share many common features. Indeed, a report by Vonaesch et al., 2018, showed that the stomach and duodenum microbiota are alike but different from the faecal microbiota (Vonaesch et al., 2018).

It is reasonable to suppose that the gastric acid environment may have a central role in the composition of gastric and duodenal microbiota. Indeed, the acid barrier protects against orally ingested microorganisms and leads to their inactivation before reaching the intestine. On this regard, the hypochloridria present in patients with chronic atrophic gastritis (CAG) might therefore represent an important cause of dysbiosis (Engstrand and Lindberg, 2013). Gastric acid environment can also be affected by *Helicobacter pylori* (*H. pylori*) colonization that, in turn, may also influence the gastric microbial community structure and composition, leading to gastric dysbiosis (Yuan et al., 2021) that may also play a role in the development of gastric adenocarcinoma (Wen et al., 2021).

The aim of this study was to assess a correlation between gastric pH and the microbiota composition, in terms of relative abundance, species diversity and phylogenetic distance, from the gastric mucosa, duodenum and gut, in patients with and without histologic gastric alterations, like CAG and non-atrophic pangastritis.

## 2. Materials and Methods

### 2.1 Patients

The patients included in the study were selected among the patients attending the Gastroenterology or Endocrinology Units of Sapienza University of Rome. The inclusion criteria were: i. adults aged between 18 and 80 years; ii. the presence of CAG under endoscopic follow-up; iii. the presence of positive predictors for CAG [current or previous history of H. pylori infection, the presence of antibodies to gastric parietal cells and/or anti-intrinsic factor, hypergastrinemia (Lahner et al., 2020), unexplained iron deficiency or pernicious anemia (Sibilla et al., 2008), thyroxine malabsorption in patients with hypothyroidism (Virili et al., 2021)]; iv. dyspepsia or gastroesophageal reflux disease (GERD) symptoms (Carabotti et al., 2017). Patients without any detection of CAG or Hp-infection during endoscopy, who underwent esophagogastroduodenoscopy (EGD) for GERD or Functional Dyspepsia (FD), were considered as control group. A standardized 7-days diet (1950 Kcal/day: carbohydrates 50%, lipids 30%, proteins 20%, fibers 25 g) was given in the week preceding the collection of biological samples to avoid bias in the composition of the gut microbiota. The exclusion criteria were: i. any contraindication for gastroscopy (e.g. severe heart failure); ii. malabsorptive or inflammatory syndromes (celiac disease, chronic intestinal inflammatory diseases, and lactose malabsorption); iii. the use of PPIs in the month prior to gastroscopy as well as the use of antibiotics or probiotics in the three months prior to gastroscopy.

All study participants gave their written informed consent prior to sampling. This study design and protocol was approved by the Umberto I University Hospital ethical committee (reference number 6160/2021) and conducted according to the principles expressed in the Declaration of Helsinki.

### 2.2 Sample Collection

From each patient were collected gastric juice for pH titration, and a faecal sample as well as 2 bioptic samples from the gastric (corpus) and duodenal mucosa (2^nd^ part) for the metagenomic analysis, and 5 bioptic samples from the gastric mucosa for the histological analysis.

At the beginning of EGD, gastric juice was collected with the aid of a sterile teflon catheter inserted in the suction channel of the gastroscope and then titrated with a 1N NaOH solution to evaluate the H+ concentration. During EGD, “sterile biopsies” using forceps for the aseptic biopsy of the mucosa with Teflon-coated catheter sealed at the distal end with a cap in silicone (Brisbane aseptic biopsy device-MTW endoskopie) were collected for the analysis of gastric and duodenal microbiota. The sterile forceps kit was changed at the time of transition between duodenum and stomach. Immediately after collection, the biopsies were placed in sterile cryotubes (ThermoFisher Scientific, US), sent to microbiology laboratory, and stored at -80°C. On the EGD day patients, each patients provided a fecal sample, that was stored at -80°C and then used for the fecal microbiota analysis.

Afterwards, according to the updated Sydney System, gastric mucosal biopsies for the histological analysis were collected as follows: two from the antrum (from the greater and lesser curvature, 3 cm from the pylorus); one from the incisura; and two from the body (from the lesser curvature, 4 cm proximal to the incisura, and from the greater curvature, middle). The histological examination was evaluated according to the Operative Link on Gastritis Assessment (OLGA), and Operative Link on Gastritis Assessment based on Intestinal Metaplasia (OLGIM) systems, currently used to stage the grade of atrophy and intestinal metaplasia (IM) (Rugge et al., 2007; Capelle et al., 2010; Rugge et al., 2012) respectively and to stratify patients according to the risk of developing gastric adenocarcinoma. Moreover, the presence of *H. pylori* was assessed by modified Giemsa stains. Histological examination was performed by a pathologist who was unaware of the clinical diagnosis of each individual patient. Fasting plasma gastrin was measured by radioimmunoassay (MP Biomedicals, USA; normal values <100 pg/ml).

### 2.3 Clinical Dyspepsia Evaluation

Patients completed a symptomatic questionnaire, a revised form of Rome IV criteria (Drossman, 2016; Drossman and Hasler, 2016) delivered on the day of the endoscopic examination. According to the current definition of the Rome IV criteria, dyspepsia was classified in epigastric pain syndrome (EPS), postprandial distress syndrome (PDS), and the overlap form of EPS/PDS. The diagnosis of dyspepsia was made in the presence of at least one of the following symptoms: epigastric burning, epigastric pain, early satiety, and postprandial fullness, regardless of fulfilling the Rome diagnostic criteria for EPS and/or PDS with symptom onset at least 6 months before diagnosis. Indeed, a score (from 0 to 3) was assigned for each item in relation to frequency and severity, and an overall clinical dyspeptic score (from 0 to 24) was obtained to subdivide dyspeptic patients in 4 groups of severity (0, absent; 1-8, mild; 9-16, moderate; 17-24, severe) (**Table 1**).

**Table 1:** Questionnaire and score system for the clinical evaluation of dyspepsia.

Score	Frequency	Severity
**0**	Absent symptoms or not diagnostic for EPS or PDS: < 3 days for month, or < 1 day a week for PDS	Absent
**1**	EPS: 1 day for week PDS: 2 days for week	Mild
**2**	EPS: > 1 day for week PDS: ≥ 3 days for week	Moderate: symptoms which do not affect activities daily
**3**	Every day	Severe: symptoms which affects activities daily

### 2.4 Microbiological Analysis

#### 2.4.1 DNA Isolation and Next-Generation Sequencing

Total DNA from gastric and duodenal biopsies was extracted using the DNeasy Blood and Tissue kit (Qiagen, USA), whereas total DNA from faecal samples was extracted using the QIAmp PowerFecal DNA kit (Qiagen, USA), according to the Manufacturer’s instructions. DNA was quantified by fluorescence spectroscopy (Quant-iT PicoGreen dsDNA Assay Kit, ThermoFisher, USA) and its integrity checked by agarose gel electrophoresis. The DNA was shipped to Bio-Fab research (Rome, Italy) for 16s rRNA gene amplification and Illumina MiSeq next-generation sequencing.

#### 2.4.2 16s rRNA Gene Amplification and Illumina MiSeq Sequencing

Dual-indexed universal primers 341F (CCTACGGGNGGCWGCAG) and 802R (GACTACHVGGGTATCTAATCC; Illumina, USA) were utilized for the two-steps PCR amplification of the V3–V4 hypervariable regions of the 16s rRNA gene (16S/ITS Nextera two-step PCR kit, Illumina Inc., USA), as previously described (Filardo et al., 2019). Briefly, the first-step PCR was carried on amplifying the V3–V4 region of the 16s rRNA gene. The resulting PCR amplicons were then used for the second-step PCR for further amplification and inclusion of indexes (barcodes) as well as the Illumina sequencing adaptors. Cycling conditions were initial denaturation at 95°C for 3 min, followed by 20 cycles (for the first-step PCR) or 15 cycles (for the second-step PCR) of denaturation at 98°C for 20 s, annealing at 56°C for 30 s and elongation at 72°C for 30 s, one cycle of final elongation at 72°C for 5 min and a final cooling step to 10°C. The resulting PCR products were quantified by fluorescence spectroscopy (Quant-iT PicoGreen dsDNA Assay Kit, ThermoFisher, USA), pooled in equimolar amounts and then purified with Agencourt AMpure-XP magnetic beads (Beckman Coulter, USA). The final library containing all the pooled samples was sequenced with version 3 of MiSeq Reagent Kit, 2 × 300 bp output, on a MiSeq desktop sequencer (Illumina, USA). One negative control (350 μL of sterile PBS) was included and subjected to the same procedures as the samples.

#### 2.4.3 Sequencing Data and Bioinformatic Analysis

MiSeq paired-end reads were subjected to demultiplexing and trimming of Illumina adaptor residuals using Illumina recommended parameter settings (Illumina MiSeq Reporter software, version 2.6). Primers were trimmed off the sequences using cutadapt (version 3.1) (Martin, 2011), and the resulting paired-end reads were then subjected to bioinformatic analysis using the software framework QIIME 2 (version 2021.2) (Bolyen et al., 2019) with the following steps: i. quality control; ii. denoising, aligning and joining of paired-end reads, as well as identification and removal of chimeric sequences, *via* the plugin dada2 (Callahan et al., 2016); iii. clustering of open-reference operational taxonomic units (OTUs) at a similarity level of 97% using the plugin v-search (Rognes et al., 2016).

The taxonomic assignment was performed by using a pre-trained naive Bayes machine-learning classifier, trained to differentiate the taxa present in the 99% Greengenes reference sequences (version 13_8), trimmed to the V3-4 hypervariable region corresponding to the primers 341F and 802R, according to the methods described by Bokulich et al., 2018 (Bokulich et al., 2018).

OTUs with only one sequence (singletons) and those not found more than 10 times in any sample were excluded from the downstream analysis to minimize artifacts. OTUs that could not be identified to a species level using the reference database, were searched using BLAST and assigned to the deepest taxonomical level based on available published data.

Taxa summaries were performed in QIIME 2, and all samples were normalized to the sample with the lowest read count for alpha and beta diversity comparisons. Shannon’s diversity and Faith’s Phylogenetic Diversity indexes were used as metrics for alpha rarefaction analysis, which was performed in QIIME 2. In particular, the Shannon’s diversity index accounts for both abundance and evenness of the species present, whereas the Faith’s phylogenetic diversity (PD) index is defined as the sum of the branch lengths of a phylogenetic tree connecting all species in the community. Jackknifed principal coordinates analyses (PCoA) was used so to assure that our rarefaction selection was not the cause of the observed clustering patterns. PCoA analysis was based on unweighted and weighted UniFrac distance matrixes and computed in QIIME 2 (Lozupone and Knight, 2005; Lozupone et al., 2007). In particular, the weighted and unweighted UniFrac analysis, the first based on sequence distances in the phylogenetic tree and on their relative abundances, whereas the latter based solely on the sequence distances. For taxa comparisons, relative abundances based on all obtained reads were used. Differential taxonomic units between groups were identified using the linear discriminant analysis (LDA) coupled with effect size measurement (LEfSe) and the Analysis of Composition of Microbiomes (ANCOM) as previously described (Segata et al., 2011; Mandal et al., 2015).

### 2.5 Statistical Analysis

Parametric data were expressed as mean ± standard deviation (SD) and were analyzed by Student’s *t*-test; the comparison between the two groups was carried out by means of the Fisher’s test.

Relative abundances of taxa were expressed as means ± standard error of means (SEM), whereas alpha diversity indexes as median (IQR). Nonparametric t test based on Monte Carlo permutations was used for alpha diversity comparisons, and Adonis was used for category comparisons of distance matrices, all calculated in QIIME 2 (Caporaso et al., 2010). The alpha-correlation analysis between pH values and alpha diversity distances was performed *via* Pearson’s product-moment correlation. Bonferroni correction was used to correct for multiple hypothesis testing when needed. The single or multiple inference significance level was set at 5%.

## 3. Results

### 3.1 Study Population Characteristics

A total of 31 patients (mean age 52.2 ± 13.1 years) were enrolled, of which 80.6% was female. 11/31 pts (35.5%) did not present any histological gastric alterations (G^0^) while 20/31 pts (64.5%) had histological gastric alterations located in the corpus and fundus (G^+^), in the absence of gastric adenocarcinoma or neuroendocrine tumor. Of them, 15/20 (75%) presented chronic atrophic gastritis (CAG), 10 of them H. pylori-related, and the remaining 5/20 (25%) presented H. pylori positive non-atrophic pangastritis. In *H. Pylori*-related CAG a current detection of histological *H. pylori* infection was present, whereas in the 5 cases of autoimmune CAG both *H. pylori* serology and biopsy were negative and only anti-parietal cells autoantibodies (APCA) were detected. In CAG patients, the Olga scores ranged between 2-3 stages and OGIM scores between 1-4. Fasting plasma gastrin serum levels in CAG patients were 692,20 ± 242,25 pg/ml. No statistically significant differences in age and BMI were present between the two groups of subjects (**Table 2**).

**Table 2:** Demographic data and histological status of the study population.

Population Characteristics	Gastric histological alterations (G^+^, n=20)	Absence of gastric histological alterations (G^0^, n=11)
M/F (%)	5 (25%)/15 (75%)	1 (9%)/10 (91%)
Age (ys) mean ± DS	52.9 ± 13.1	51 ± 13.5
BMI (kg/m^2^) mean ± DS	23.7 ± 4.2	22.6 ± 2.8

### 3.2 pH Determination in Gastric Juice

Intragastric pH, in G^+^ patients (5.89 ± 2.30), was statistically higher than pH values in G^0^ patients (2.10± 1.7, *p*<0.001). Besides, among G^+^ patients, pH in CAG patients was 6.82 ± 1.43, statistically higher than in H. pylori+ pangastritis (3.09 ± 2.23; *p*=0.0003).

### 3.3 Evaluation of Clinical Dyspeptic Symptoms in the Study Population

Dyspepsia was detected in 72.7% of G^0^ patients (8/11) and in 55.5% (10/18) of G^+^ patients, due to the drop out of 2 patients with Hp+ pangastritis in filling up the symptomatic questionnaire. In CAG, dyspepsia was present in 40% of the patients (6/15) [40% of Hp+ CAG (4/10), 60% autoimmune CAG (3/5)]. Considering the whole group of Hp+ patients, dyspepsia was present in 66.7% of the patients (7/13).

G0 patient have an average total dyspeptic score of 9.37 ± 6.84, higher than that of the G^+^ patients (5.90 ± 2.99). In CAG, the total dyspeptic score was 5.14 ± 2.41, whereas, in the whole group of Hp+ patients, was 6.86 ± 2.67.

Specifically, among dyspeptic G^+^ patients, 2 suffer from PDS, 2 from PDS/EPS, whereas 6 have low symptoms frequency that did not allow a diagnosis of EPS or PDS. A similar distribution of dyspepsia subtypes was found in G^0^ patients, 3 being affected by PDS, 2 by PDS/EPS, while 6 have low symptoms frequency that did not allow a diagnosis of EPS or PDS.

### 3.4 Gastric, Duodenal, and Faecal Microbiota Composition

An average of 2848 [median (Interquartile Range, IQR) 2726 (1671)], 3703 [2479 (3134)] and 60632 [6041 (117984)] paired-end Illumina reads were analyzed in gastric, duodenal, and faecal samples, respectively by metagenomic analysis of the hypervariable region V3-4 from the bacterial 16s rDNA *via* Illumina next generation sequencing. After the removal of singletons and rare Operational Taxonomic Units (OTUs), a total number of 162 [46 (18)], 154 [39 (34)] and 552 [73 (63)] OTUs were identified in gastric, duodenal, and faecal samples, respectively. The lowest read count was 1257 for gastric biopsies, 931 for duodenal biopsies and 2935 for faecal samples; hence, OTUs were randomly subsampled to 1257, 931 and 2935 reads, respectively, for diversity analysis to avoid further bias. As expected, the total number of reads as well as OTUs was significantly higher in faecal samples as compared to gastric or duodenal biopsies (*p*=0.000003 and *p*=0.00025, respectively, for the reads, and *p*=0.0001 and *p*=0.000019, respectively, for the OTUs), whereas the gastric and duodenal mucosa showed similar sequencing results.

As a first step, we analyzed the gastric, duodenal and faecal microbiota composition of G^+^ patients as compared to G^0^ patients. As shown in **Table 3**, the gastric microbiota did not differ between these two groups, and only a slight increase in the relative abundance of the phylum Firmicutes and a decrease in the relative abundances of the phyla Bacteroidetes and Actinobacteria were observed in G^+^ patients as compared to G^0^ patients. Instead, significant differences were observed concerning the duodenal microbiota. Indeed, in G^+^ patients, a statistically significant increase in the relative abundance of the phyla Firmicutes (*p*=0.02), Fusobacteria (*p*=0.027) and Actinobacteria (p=0.036) as well as to a decrease in the relative abundance of the phyla Proteobacteria (*p*=0.047) and Tenericutes (*p*=0.01) were observed as compared to G^0^ patients. A similar trend was observed for the faecal microbiota where a slight increase in the relative abundance of the phyla Firmicutes, Proteobacteria and Bacteroidetes could be observed in G^+^ compared to G^0^ patients.

**Table 3:** Relative abundances of stomach, duodenal and faecal microbiota composition in G^0^ and G^+^ patients.

	Stomach		Duodenum		Fecal	
	G^+^ patients	G^0^ patients	G^+^ patients	G^0^ patients	G^+^ patients	G^0^ patients
**Firmicutes**	37.31 ± 2.32	29.64 ± 3.61	34.71 ± 3.57*	18.76 ± 1.48	76.37 ± 8.23	63.65 ± 16.68
**Proteobacteria**	30.28 ± 1.50	32.32 ± 2.67	30.34 ± 2.77	49.23 ± 3.10*	6.73 ± 0.77	1.80 ± 0.36
**Bacteroidetes**	21.35 ± 1.11	25.54 ± 2.79	23.41 ± 2.43	22.44 ± 2.15	15.46 ± 1.95	12.09 ± 2.57
**Fusobacteria**	4.54 ± 0.31	3.75 ± 0.50	5.48 ± 0.64*	1.72 ± 0.43	0.05 ± 0.01	0.01 ± 0.00
**Actinobacteria**	4.71 ± 0.33	6.35 ± 0.99	4.52 ± 0.46*	2.51 ± 0.33	1.12 ± 0.14	0.98 ± 0.28
**Aquificae**	0.68 ± 0.11	0.32 ± 0.10	0.76 ± 0.16	0.97 ± 0.43	0.00 ± 0.00	0.00 ± 0.00
**Tenericutes**	0.90 ± 0.06	1.61 ± 0.18	0.65 ± 0.06	4.38 ± 0.41*	0.00 ± 0.00	0.01 ± 0.00
**Spirochaetes**	0.19 ± 0.03	0.42 ± 0.13	0.12 ± 0.02	0.00 ± 0.00	0.00 ± 0.00	0.00 ± 0.00
**Verrucomicrobia**	0.00 ± 0.00	0.00 ± 0.00	0.02 ± 0.01	0.00 ± 0.00	0.13 ± 0.02	0.57 ± 0.19
**Cyanobacteria**	0.04 ± 0.01	0.04 ± 0.02	0.00 ± 0.00	0.00 ± 0.00	0.03 ± 0.01	0.02 ± 0.01
**Synergistetes**	0.00 ± 0.00	0.00 ± 0.00	0.00 ± 0.00	0.00 ± 0.00	0.11 ± 0.02	0.00 ± 0.00
**Lentisphaerae**	0.00 ± 0.00	0.00 ± 0.00	0.00 ± 0.00	0.00 ± 0.00	0.01 ± 0.00	0.00 ± 0.00

Only taxa with abundances greater than 0.01% in any sample were included. Data are expressed as mean ± SE; *p<0.05 vs G^0^.

To assess the possible influence of pH in the observed microbiota differences in G^+^ patients, the analysis was performed comparing the sole patients with CAG, who had a low-acid environment, to G^0^ patients, who possessed a normal acid gastric environment. Similar to what previously described, most differences were observed in the duodenal microbiota, with CAG patients presenting a statistically significant increase, in respect to G^0^ patients, in the relative abundance of the phyla Firmicutes (35.16 ± 3.9 vs 18.76 ± 1.48, *p*=0.032) and Actinobacteria (4.69 ± 0.5 vs 2.51 ± 0.33, *p*=0.01), and a decrease in the relative abundance of the phyla Proteobacteria (30.21 ± 2.97 vs 49.23 ± 3.1, *p*=0.02) and Tenericutes (0.55 ± 0.06 vs 4.38 ± 0.41, *p*=0.027). By contrast, the gastric and faecal microbiota did not significantly differ between CAG patients and G^0^ patients.

### 3.5 Alpha- and Beta- Diversities Analysis

Initially, we investigated whether there were differences in the species diversities of the microbial communities observed in each anatomical site in all patients (gastric and duodenal mucosa, and faecal samples) *via* measures of alpha- and beta- diversities, namely Shannon’s diversity index and Faith’s phylogenetic diversity, as well as the weighted and unweighted UniFrac analysis.

**Figure 1:**
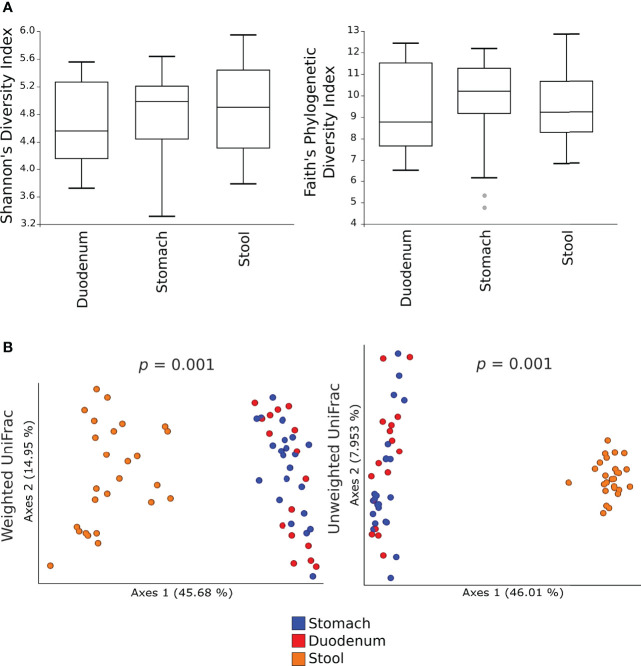
Alpha- and beta- diversity of the microbial communities in gastric, duodenal, and faecal samples. Shannon’s diversity and Faith’s phylogenetic diversity indexes were used as measure of alpha-diversity within groups **(A)**. Samples were rarefied to the smallest observed number of reads (1257 for gastric biopsies, 931 for duodenal biopsies and 2935 for faecal samples). The circles out of the range represent the outliers; Principal coordinate analysis (PCoA) plots of weighted and unweighted UniFrac distances are represented **(B)**. Each dot represents the bacterial community composition of one individual at each anatomical site. Groups were compared using Adonis for beta diversity.

Alpha-diversity measures showed no significant differences in the microbial communities between gastric, duodenal, and faecal samples (**Figure 1A**). By contrast, both the weighted and unweighted UniFrac analyses demonstrated a statistically significant clustering of microbial communities from faecal samples as compared to gastric or duodenal mucosal biopsies (p=0.001), suggesting shifts in relative abundances as well as phylogenetic distances of species composition (**Figure 1B**). These results highlight the similarity of the microbiota in the gastric and duodenal mucosa, without significant variations between the two anatomical sites, whereas the faecal microbiota is significantly distant in both relative abundance and species composition.

**Figure 2:**
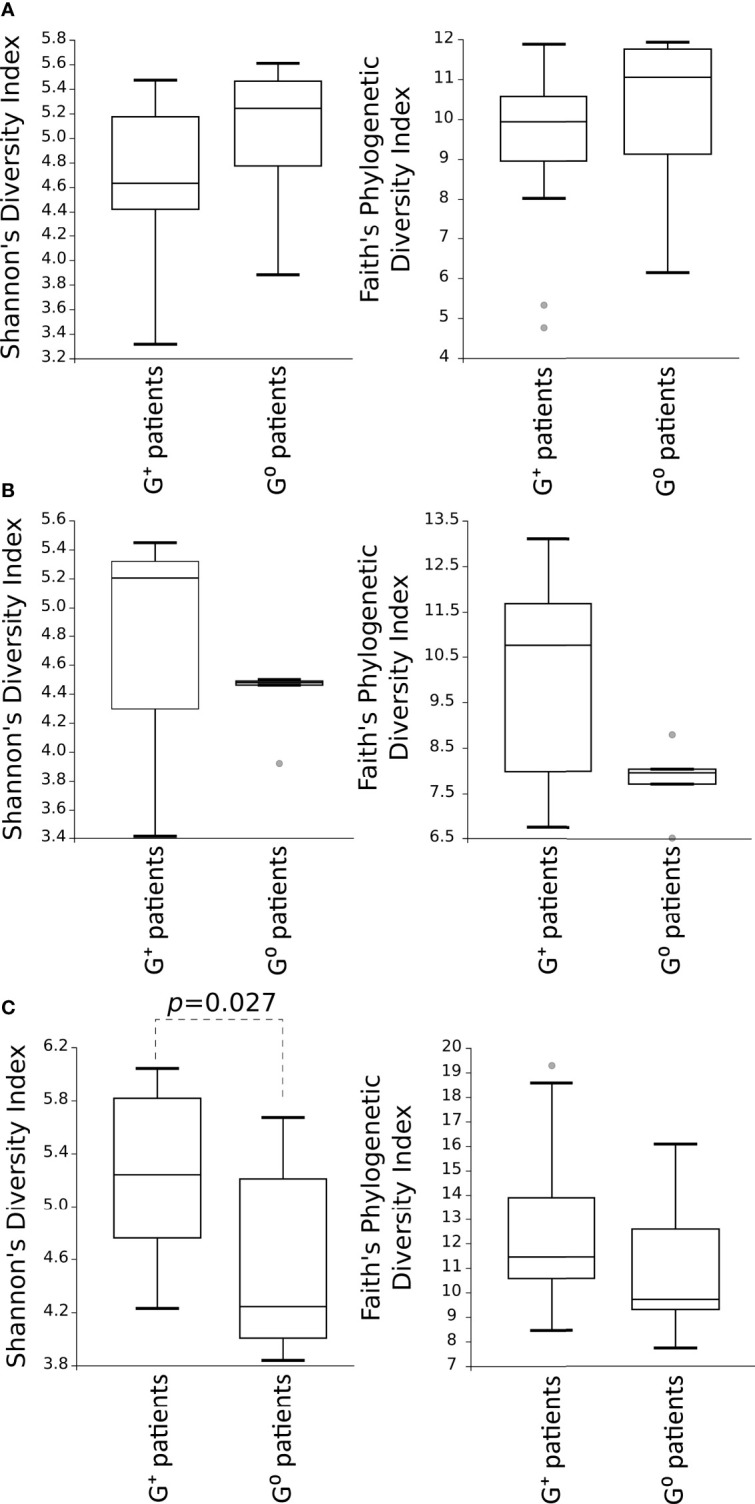
Comparison of the alpha-diversity of the microbial communities between G^+^ and G^0^ patients. Microbiota from gastric **(A)** and duodenal **(B)** biopsies, as well as from faecal **(C)** samples, are represented. Shannon’s diversity and Faith’s phylogenetic diversity indexes were used as measure of alpha-diversity within groups. Samples were rarefied to the smallest observed number of reads (1257 for gastric biopsies, 931 for duodenal biopsies and 2935 for faecal samples). The circles out of the range represent the outliers.

Then, alpha-diversity measures of the microbial communities in G^+^ patients were compared to G^0^ patients, showing no differences for both Shannon’s diversity and Faith’s phylogenetic diversity indexes in either the gastric or duodenal microbiota (**Figures 2A, B**). Of note, is the trend of low variability of duodenal microbiota community in G^0^ patients. In the faecal microbiota, Shannon’s diversity index evidenced a higher species diversity in G^+^ than in G^0^ patients (p=0.027) (**Figure 2C**).

**Figure 3:**
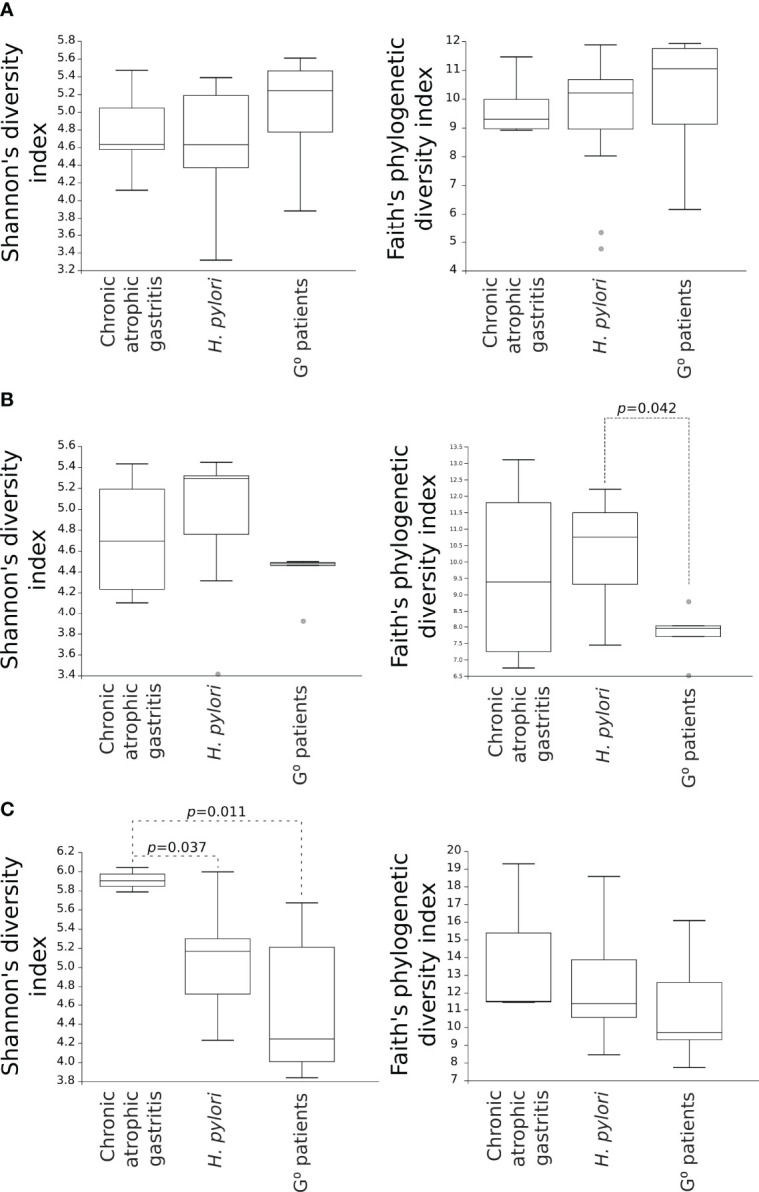
Comparison of alpha-diversity indexes of microbial communities amongst CAG, *H. pylori* non-atrophic pangastritis and G^0^ patients. Microbiota from gastric **(A)** and duodenal **(B)** biopsies, as well as from faecal **(C)** samples, are represented. Shannon’s diversity and Faith’s phylogenetic diversity indexes were used as measures of alpha-diversity within groups. Samples were rarefied to the smallest observed number of reads (1257 for gastric biopsies, 931 for duodenal biopsies and 2935 for faecal samples). .

Considering the alpha-diversity in each gastric condition independently, the Shannon’s as well as Faith’s phylogenetic diversity indexes of the duodenal microbiota were higher for both CAG and H. pylori non-atrophic patients as compared to G^0^ patients, although only the Faith’s phylogenetic diversity of the duodenal microbiota in H. pylori non-atrophic patients reached statistical significance (p=0.042, **Figure 3B**). In the faecal microbiota, only the presence of CAG contributed to a significantly higher species diversity as compared to both H. pylori non-atrophic and G^0^ patients (p=0.037 and p=0.011, respectively, **Figure 3**).

**Figure 4:**
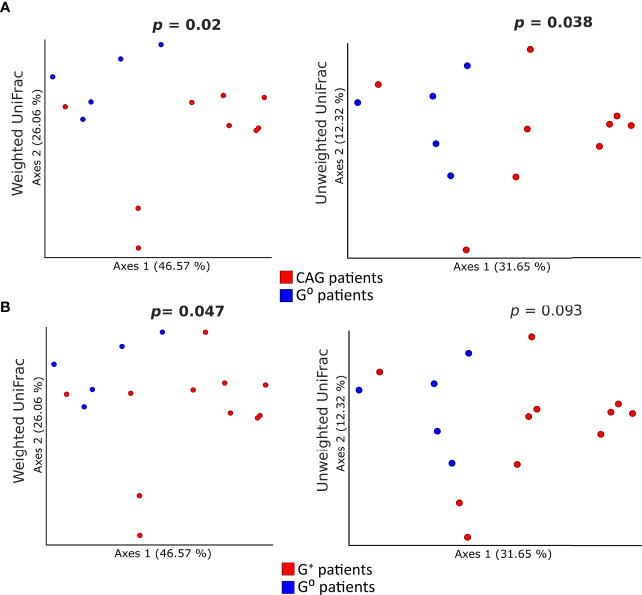
Comparison of the beta-diversity of duodenal microbiota between CAG or G^+^ patients, and G^0^ patients. Principal coordinate analysis (PCoA) plots of weighted and unweighted UniFrac distances of the microbiota from duodenal biopsies of CAG **(A)** and G^+^ patients **(B)** are illustrated. Each dot represents the bacterial community composition of one individual at each anatomical site. Groups were compared using Adonis for beta diversity.

Concerning the beta-diversity measures, a statistically significant clustering of bacterial communities from the duodenal microbiota of CAG patients as compared to G^0^ patients (**Figure 4A**) was evidenced both in the weighted (*p*=0.02) and unweighted (*p*=0.038) UniFrac analysis. By contrast, in G^+^ patients, only the weighted UniFrac analysis of the duodenal microbiota showed a tendency toward a statistically significant clustering, as compared to G^0^ patients (*p*=0.047) (**Figure 4B**). No clustering in the gastric and faecal microbiota was observed when comparing either weighted or unweighted UniFrac distance matrixes of CAG patients (*p*=0.57, *p*=0.82, *p*=0.14 and *p*=0.44, respectively) or G^+^ patients (*p*=0.24, *p*=0.58, *p*=0.82 and *p*=0.89, respectively), to G^0^ patients.

### 3.6 Correlation of Microbiota Diversity With Gastric pH

**Figure 5:**
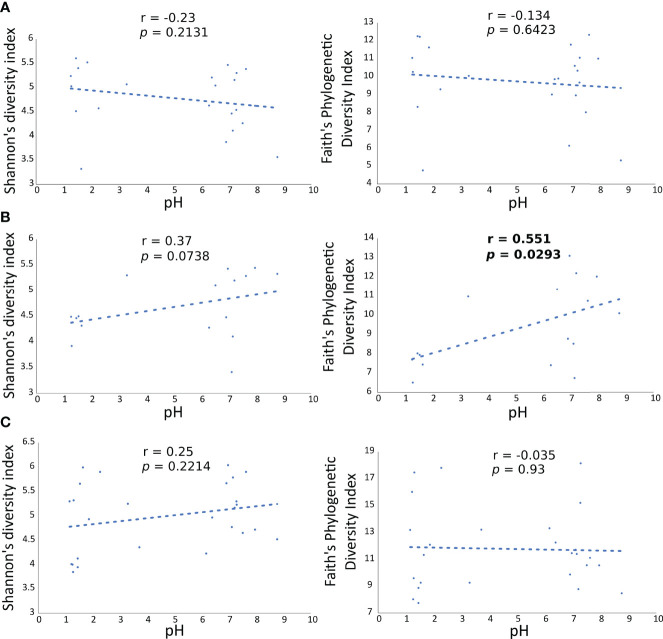
Correlation of Faith’s PD diversity index with pH values in all patients at each anatomical site. Each dot is the gastric **(A)** duodenal **(B)** and faecal **(C)** microbiota of a patient, represented as alpha diversity on the y axes, related to the respective pH value, on the x axes.

To validate the influence of pH on the diversity of the microbial communities in the gastric and duodenal mucosa, as well as in the faecal samples, Shannon’s and Faith’s phylogenetic diversities indexes of each microbial community were correlated to the pH values of individual patients *via* Pearson’s product-moment correlation (**Figure 5**). A statistically significant increase in Faith’s phylogenetic diversity index at increasing pH values was observed only in the duodenal microbiota (*p*=0.0293, **Figure 5B**), whereas the diversity of gastric (**Figure 5A**) and faecal microbiota (**Figure 5C**) were not affected by the increment in pH.

### 3.7 Association of Specific Taxonomic Units to G^+^, CAG or G^0^ Patients

To identify specific taxa in gastric and duodenal biopsies, as well as faecal samples, associated to either G^+^, CAG or G^0^ patients, 2 different approaches, namely the linear discriminant analysis (LDA) coupled with effect size measurement (LEfSe) and the Analysis of Composition of Microbiomes (ANCOM) were used. While the analysis of gastric and faecal microbiota did not reveal any specific correlations between taxonomic units and gastric conditions, an association with defined OTUs was observed in the duodenal microbiota community.

**Figure 6:**
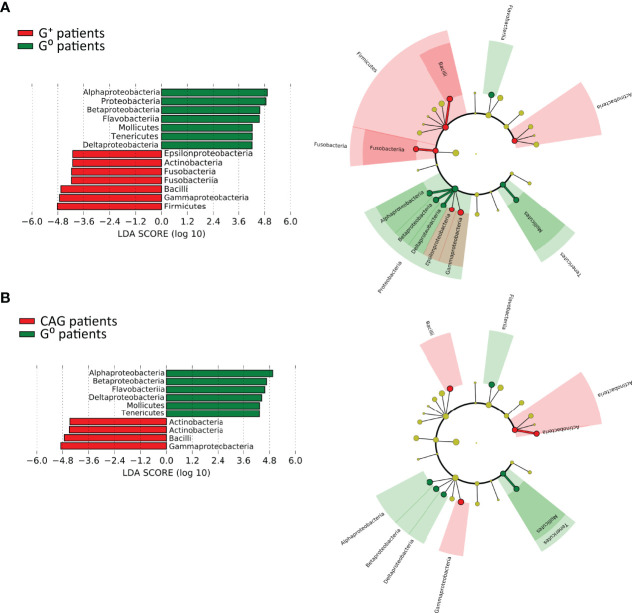
Linear discriminant analysis with effect size measurement (LEfSe) of the duodenal microbiota between CAG or G^+^, and G^0^ patients. LEfSe analysis in CAG **(A)** and G^+^ patients **(B)**. On the left, histogram of the LDA scores computed for statistically significant differentially abundant taxa between the two groups. On the right, cladogram highlighting the relationship of significantly different taxa between G+ and G0 patients. Differences are represented in the color of the most abundant class, and each circle’s diameter is proportional to the taxon’s abundance.

In particular, the LEfSe analysis for phyla/genera in G^+^ patients (**Figure 6A**) identified a strong association of the phyla Firmicutes (specifically the class Bacilli, including the genera *Granulicatella* and *Streptococcus*, LDA > 5.0, *p*=0.02), Fusobacteria (specifically the class Fusobacteriia, including the genera *Leptotrichia* and *Streptobacillus*, LDA > 4.5, *p*=0.027), Actinobacteria (specifically the order Actinomycetales, including the genus *Rothia*, LDA > 4.5, *p*=0.036) as well as Proteobacteria (specifically Epsilonproteobacteria and Gammaprotebacteria, including the genera *Campylobacter* and *Actinobacillus*, LDA > 4.0, *p*=0.02). Interestingly, only the Gammaproteobacteria (LDA > 4.5, *p*=0.0036), Actinobacteria (LDA > 4.0, *p*=0.01) and Firmicutes (LDA > 4.5, *p*=0.032) were associated to CAG patients with the lower-acid environment (**Figure 6B**). By contrast, G^0^ patients were associated to the phyla Bacteroidetes (specifically the class Flavobacteriia, including the genus *Sulcia*, LDA > 5.0, *p*=0.006), Proteobacteria (specifically the class Alphaproteobacteria, Betaproteobacteria and Deltaproteobacteria, including the genera *Caulobacter*, *Phenylobacterium*, *Herbaspirillum* and *Desulfatiglans*, LDA > 5.0, *p*=0.047) and Tenericutes (specifically the class Mollicutes, including the genus *Spiroplasma*, LDA > 4.0, *p*=0.011) (**Figure 6**).

**Figure 7:**
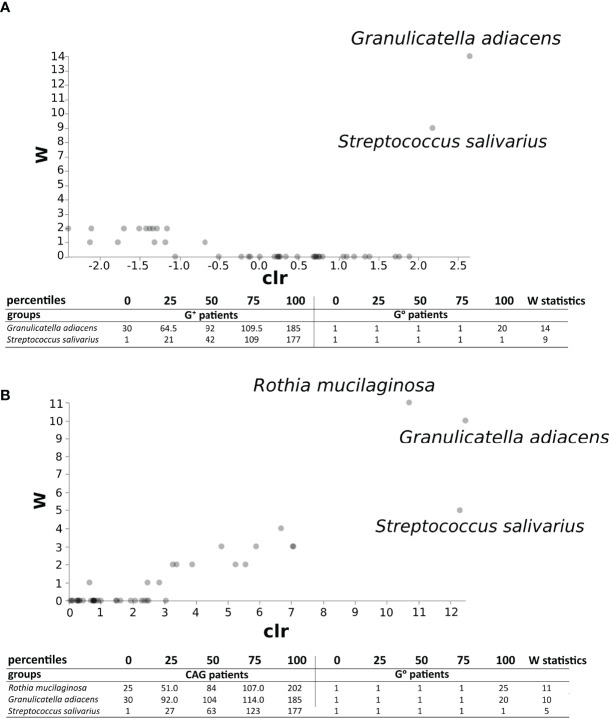
ANCOM test in the duodenal microbiota between CAG or G+ patients, and G^0^ patients. ANCOM analysis in CAG **(A)** and G^+^ patients **(B)**. W statistics represent the number of times the null hypothesis is rejected for a given taxon.

The ANCOM test (**Figure 7A**) evidenced, in the duodenal microbiota, the significant association of bacterial species of oral origin, namely *Granulicatella adiacens* and *Streptococcus salivarius*, belonging to the phylum Firmicutes, to G^+^ patients (W statistic 14 and 9, respectively). Notably, when only CAG patients were considered, a further oral bacterial specie resulted significantly associated, namely *Rothia mucilaginosa* (W statistic 11, **Figure 7B**).

## 4. Discussion

To our knowledge, this is the only study aimed to correlate the mucosal-associated microbiota of the stomach and duodenum to the intragastric pH of the same patients with and without histologic gastric alterations, like CAG and non-atrophic pangastritis (Gong et al., 2019) Indeed, none of the previous studies directly quantified intragastric pH, but indirectly assumed the presence of hypochlorhydria either from the use of PPIs, from the positivity to *H. pylori* infection, or from the diagnosis of CAG. However, because the intragastric pH can vary substantially under these conditions, in the present study, all patients underwent histological examination of the gastric and duodenal mucosa along with the measurement of intragastric pH, excluding potential bias related to the diet and the use of PPIs.

In accordance with literature data, in our study the gastric microbiota composition was similar to the duodenal one, in term of beta diversity measures, and both differed from the fecal microbiota (Vonaesch et al., 2018). However, despite the similarities, stomach and duodenum differently reacted to hypochlorhydria, and the duodenum seemed to be the site that was mostly affected by pH modifications. Indeed, the main result of the present work is the direct correlation of duodenal microbiota biodiversity, *via* Faith’s phylogenetic diversity index, a measure of alpha diversity, with intragastric pH values. Specifically, patients with hypochlorhydria, namely chronic atrophic gastritis with the lower-acid environment, showed increased duodenal microbiota biodiversity. Hints of a correlation between duodenal microbiota and intragastric pH was also suggested by a recent study, observing an elevated mucosal bacterial load in the proximal small intestine of patients treated with PPIs, usually causing hypochlorhydria, although they did not investigate species composition and diversity (Shah et al., 2020).

A further interesting data is the statistically significant clustering of the duodenal microbiota composition in relation to the presence of histologic gastric alterations, in particular chronic atrophic gastritis, as evidenced by UniFrac analysis, a measure of beta-diversity. By contrast, alpha-diversity measures of the duodenal as well as gastric microbiota did not significantly differ between patients with or without histologic gastric alterations. However, in our study, a decreased diversity of the gastric microbiota, and an increased diversity of the duodenal microbiota were still observed in relation to the presence of CAG, although they did not reach statistical significance. Interestingly, the only condition that independently reduced the gastric microbiota diversity was the presence of *H. pylori* infection (**Figure 3**), data also confirmed by other studies (Andersson et al., 2008; Parsons et al., 2017; Bravo et al., 2018). We also demonstrated that in patients with histologic gastric alterations the faecal microbiota showed increased alpha-diversity, similar to the duodenal microbiota, suggesting the possibility that the faecal microbiota could potentially mirror the duodenal microbiota. Overall, it can be hypothesized that in a normal intragastric acidic environment, associated to the absence of histologic alterations, duodenal eubiosis is characterized by a low microbial load and decreased microbiota diversity, while their increase corresponds to duodenal dysbiosis. As a result, the presence of duodenal dysbiosis may lead to the development of pathological conditions of the gastrointestinal tract. For example, the presence of microbiota dysbiosis, as well as increased microbial load, were correlated to SIBO (small intestinal bacterial overgrowth), a condition associated with numerous gastrointestinal disorders, mainly weight loss, diarrhea, bloating and malabsorption (Leite et al., 2020). The hypochlorhydria deriving from the chronic use of PPIs has been considered as a possible cause for the onset of SIBO (Spiegel et al., 2008; Lo and Chan, 2013); of note, as evidenced by our data, hypochlorhydria, linked to the presence of chronic atrophic gastritis, is able to affect the composition of the duodenal microbiota, eventually leading to gastrointestinal disorders, including SIBO.

In our study, no correlation between gastric, duodenal, or fecal microbiota diversity and composition, and the presence and burden of dyspeptic symptoms, was observed, and, hence, the clinical impact of microbiota alterations occurring in hyposecretory gastric conditions remains to be clarified. However, recent evidence has suggested that disturbances of the gastroduodenal microbiota may also be implicated in the pathogenesis of dyspepsia, one of the most prevalent upper gastrointestinal syndromes (Paroni Sterbini et al., 2016; Igarashi et al., 2017; Zhong et al., 2017; Fukui et al., 2020; Tziatzios et al., 2020; Cervantes et al., 2021).

As for the species composition, our results showed a strong association of specific taxonomic units with the duodenal microbiota of patients with histologic gastric alterations, especially patients with a low-acid gastric environment. In particular, Firmicutes, Actinobacteria and some Proteobacteria classes (e.g. Gammaproteobacteria) were most prevalent in these patients, as compared to those without alterations. Importantly, the presence of *Streptococcus salivarius* and *Granulicatella adiacens* in the duodenal microbiota was strongly associated to the presence of histologic gastric alterations. More importantly, the presence of another oral bacteria, *Rothia mucilaginosa*, detected when only the patients with CAG were analyzed, suggested that a low-acid gastric environment offers a hospitable habitat, making these oral commensals resident elements of the duodenal flora. Indeed, as evidenced in the literature, the gastric microbiota was demonstrated to be enriched of oral taxa (Coker et al., 2018), such as *Streptococcus*, *Staphylococcus* and *Lactococcus*, in the different gastric precancerous states, including CAG (Parsons et al., 2017; Coker et al., 2018), although data are still contradictory (Ferreira et al., 2018).

The main strength of our study is the application of strict inclusion criteria, including controlled diet, absence of PPIs usage, no antibiotic or probiotic treatment prior to gastroscopy, etc., that allowed us to greatly diminish the impact of the confounding bias associated to the selection of the study population leading, unfortunately, to a small sample size. In addition to fecal samples, the investigation of biopsies from different anatomical sites, namely the stomach and the duodenum, collected *via* sterile forceps to reduce potential cross contamination, represents a further important strength. However, the small sample size derived from the application of the strict inclusion criteria, does not allowed in the present study to evaluate specific microbiological differences directly related to *H. pylori*, as previously reported on gastric microbiota (Parsons et al., 2017).

In conclusion, our results suggest a low-acid gastric environment as a contributive factor for duodenal dysbiosis, opening the way to further research investigating the intriguing interplay between hypochlorhydria and microbiota in the development of the pathological conditions of the gastrointestinal tract.

## Data Availability Statement

The datasets presented in this study can be found in online repositories. The names of the repository/repositories and accession number(s) can be found below: https://www.ncbi.nlm.nih.gov/, PRJNA795512.

## Ethics Statement

The studies involving human participants were reviewed and approved by Umberto I University Hospital ethical committee (reference number 6160/2021). The patients/participants provided their written informed consent to participate in this study.

## Author Contributions

MC, RS, and CS designed and supervised this study. GS, GBr, and SP conducted upper endoscopy examinations. SF, GS, CV, SP, AC, GBr, PM, IS, MC, and CS contributed to subject recruitment and GS, CV, SP, AC, PM, and GBe contributed to sample collection. GS, SP, AC, GBe, and CS completed histopathological diagnoses. SF and MDP performed DNA extraction and metagenomic analysis. GS, SF, MP, RS, and CS wrote the first draft of the manuscript. All authors revised the manuscript and approved the submitted version.

## Conflict of Interest

The authors declare that the research was conducted in the absence of any commercial or financial relationships that could be construed as a potential conflict of interest.

## Publisher’s Note

All claims expressed in this article are solely those of the authors and do not necessarily represent those of their affiliated organizations, or those of the publisher, the editors and the reviewers. Any product that may be evaluated in this article, or claim that may be made by its manufacturer, is not guaranteed or endorsed by the publisher.
